# Global latitudinal gradients and the evolution of body size in dinosaurs and mammals

**DOI:** 10.1038/s41467-024-46843-2

**Published:** 2024-04-05

**Authors:** Lauren N. Wilson, Jacob D. Gardner, John P. Wilson, Alex Farnsworth, Zackary R. Perry, Patrick S. Druckenmiller, Gregory M. Erickson, Chris L. Organ

**Affiliations:** 1 University of Alaska Museum, 1962 Yukon Drive, Fairbanks, AK 99775 USA; 2 Department of Geosciences, University of Alaska, Fairbanks, AK 99775 USA; 3 School of Biological Sciences, University of Reading, Reading, RG6 6EX UK; 4 Department of Earth Sciences, Montana State University, Bozeman, MT 59715 USA; 5 School of Geographical Sciences, University of Bristol, University Road, Bristol, BS8 1RL UK; 6 State Key Laboratory of Tibetan Plateau Earth System, Resources and Environment (TPESRE), Institute of Tibetan Plateau Research, Chinese Academy of Sciences, Beijing, 100101 China; 7 Department of Biological Science, Florida State University, Tallahassee, FL 32306 USA

**Keywords:** Palaeontology, Macroecology, Phylogenetics, Animal physiology

## Abstract

Global climate patterns fundamentally shape the distribution of species and ecosystems. For example, Bergmann’s rule predicts that homeothermic animals, including birds and mammals, inhabiting cooler climates are generally larger than close relatives from warmer climates. The modern world, however, lacks the comparative data needed to evaluate such macroecological rules rigorously. Here, we test for Bergmann’s rule in Mesozoic dinosaurs and mammaliaforms that radiated within relatively temperate global climate regimes. We develop a phylogenetic model that accounts for biases in the fossil record and allows for variable evolutionary dispersal rates. Our analysis also includes new fossil data from the extreme high-latitude Late Cretaceous Arctic Prince Creek Formation. We find no evidence for Bergmann’s rule in Mesozoic dinosaurs or mammaliaforms, the ancestors of extant homeothermic birds and mammals. When our model is applied to thousands of extant dinosaur (bird) and mammal species, we find that body size evolution remains independent of latitude. A modest temperature effect is found in extant, but not in Mesozoic, birds, suggesting that body size evolution in modern birds was influenced by Bergmann’s rule during Cenozoic climatic change. Our study provides a general approach for studying macroecological rules, highlighting the fossil record’s power to address longstanding ecological principles.

## Abstract (web summary)

Bergmann’s Rule predicts larger body sizes in colder climates. Here, the authors examine extinct and extant dinosaurs (birds) and mammaliaforms, finding no evidence of body size variation with latitude in any group, but a small variation with temperature in extant birds.

## Introduction

Macroecological rules provide vital insights into the structure and function of ecosystems across geologic time^1^, and aid in conservation and management decisions^2^. For instance, Bergmann’s rule predicts that homeothermic animals from cooler (higher latitude) climates are generally larger than close relatives from warmer (lower latitude) climates^3–5^. Initially proposed for mammals as an adaptation for homeothermic heat retention, the rule has also been applied to birds^6,7^, but also to poikilotherms, including amphibians^8^, reptiles^9^, fishes^10^, and invertebrates^11–13^, where the inverse pattern is occasionally observed. However, there is disagreement about how the rule operates^4,5^, and its application across geologic timescales remains unclear^14^, thus hindering inferences about the ecophysiology of extinct organisms^15^ and the evolutionary responses to anthropogenic climate change^16–18^.

A strength of macroecological rules is that their hypotheses yield clear predictions^4,5^ that can be tested with phylogenetically-informed statistical models^19^. Research testing the predictions of Bergmann’s rule has, however, been hampered by three problems. First, it is common to find examples of taxa that fit Bergmann’s rule by subsampling larger datasets at varying taxonomic levels^4,5^. This is a serious problem because any sufficiently large dataset can be arbitrarily subdivided into groups, each of which may show a trend. Moreover, Bergmann’s rule is a “rule” precisely because it is hypothesised to apply across homeotherms generally (e.g., birds and mammals), not to a select few subgroups. Second, ecological rules like Bergmann’s require a model that allows the evolutionary rate of biogeographical dispersal and body size to vary across lineages^20^. Such an approach can detect differences between close relatives (immediate descendants from an ancestor) predicted by the rule. Third, and perhaps most importantly, macroecological rules often lack null models because they are hypothesised to operate broadly (e.g., across Mammalia) where natural controls are limited^21^. However, the fossil record provides repeated “natural experiments” across geological time that can be used to test general ecological rules. Despite this, research on Bergmann’s rule has focused, with rare exceptions^14,22^, on extant biodiversity and present-day climatic patterns. Mesozoic dinosaurs and mammaliaforms are an ideal contrast for studying Bergmann’s rule because they are ancestral to the two major extant homeothermic groups, birds and mammals, and inhabited a more broadly temperate world than the Present^23,24^. Moreover, dinosaurs dispersed globally and persisted for over 170 million years^25^, during which they evolved body sizes from several kilograms to over 50 tonnes^26^. Mesozoic mammaliaforms represent a second, phylogenetically distinct clade that independently radiated globally under the same climate regimes.

Here, we test for Bergmann’s rule under less-extreme global temperature gradients using data for 62 Mesozoic mammaliaforms and 339 dinosaurs, including the latest data on high-latitude dinosaur fossils from the Late Cretaceous Prince Creek Formation of northern Alaska. We assess whether body size coevolved with palaeogeographic dispersal and palaeotemperature while accounting for fossil record biases^27^ and use models that capture evolutionary rate variation^20^. We then apply our approach to large datasets of extant birds and mammals, where Bergmann’s rule would have important implications for how ecosystems are structured along latitudinal climatic gradients^28^.

## Results

### Bergmann’s rule in Mesozoic dinosaurs and mammals

Bergmann’s rule predicts that evolutionary increases in body size can be explained by positive shifts in absolute latitude and associated decreases in local climatic temperature along phylogenetic lineages. To verify our phylogenetic approach, we simulated a correlated evolution model under the expectations of Bergmann’s rule and provide a positive test case with ursids, as well as a negative control (Supplementary Fig. 1).

To assess Bergmann’s rule under less extreme global climate regimes, we regressed the femoral circumferences (a body size proxy^29^, log_10_ millimetres) of 339 Mesozoic dinosaurs onto palaeolatitude (absolute values – distance from the equator). Because climatic conditions varied across the Mesozoic, our model accounts for differences in geologic period (Triassic, Jurassic, and Cretaceous). To account for geographic range, our model implements a Bayesian reversible-jump Markov-chain Monte Carlo (RJMCMC) procedure to randomly sample latitudes for 120 Mesozoic dinosaur species (including several members of Avialae) found in multiple localities. Our models also account for these additional factors: differences between hemispheres, fossil record bias (see below), and lineages (clades). Model selection using Bayes factors (BF) favours the simplest model without additional factors (BF = 13.4–57.54, where BF > 2.0 indicates positive evidence; Supplementary Table 1). While few studies have applied variable-rate phylogenetic models to Bergmann’s rule^30^, we also find considerable support for variable rates of body size evolution with respect to latitudinal dispersal (BF = 71.41; Supplementary Table 3). Our final model accounting for variable rates shows no relationship between body size and palaeolatitude among dinosaurs (*p*_MCMC_ = 0.13, median β = 0.0009 (95% CI = −0.0007, 0.002), median *R*^2^ = −0.039 (95% CI = −0.059, −0.02); Supplementary Table 3, Supplementary Fig. 2).

**Fig. 1:**
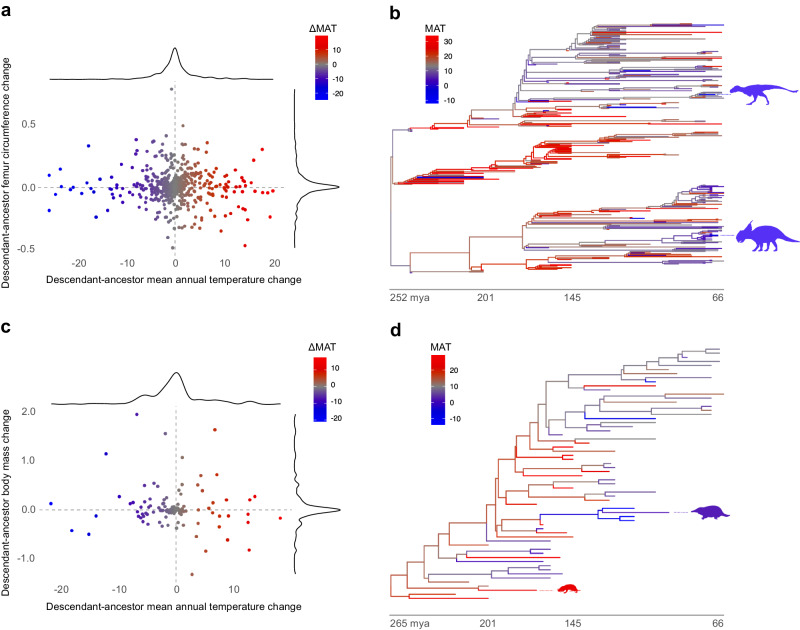
The evolution of body size in Mesozoic dinosaurs and mammaliaforms is not explained by palaeotemperature. **a** Estimated changes in femur circumference (log_10_ mm) as a function of changes in mean annual temperature (°C) along branches of the dinosaur phylogeny. The central intersection of dotted lines indicates no evolution in either trait. A trend from upper left to lower right would be consistent with Bergmann’s rule. Point colour reflects estimated changes in MAT. **b** Mesozoic dinosaur phylogeny with branches mapped by MAT. Time scale in millions of years. Silhouettes highlight *Nanuqsaurus* (Jaime Headden; CC BY 3.0) and *Pachyrhinosaurus* (Andrew A. Farke; CC BY 3.0) of the high-latitude Cretaceous Prince Creek Formation, coloured by estimated MAT. **c** Estimated changes in body mass (log_10_ grams) as a function of changes in MAT (°C) along branches of the Mesozoic mammaliaform phylogeny. **d** Mesozoic mammaliaform phylogeny with branches mapped by MAT. Silhouettes highlight *Morganucodon* (Michael B. H.; CC BY-SA 3.0) and *Steropodon* (Nobu Tamura, vectorized by T. Michael Keesey; CC BY 3.0), coloured by estimated MAT. MAT mean annual temperature, mya million years ago.

**Fig. 2:**
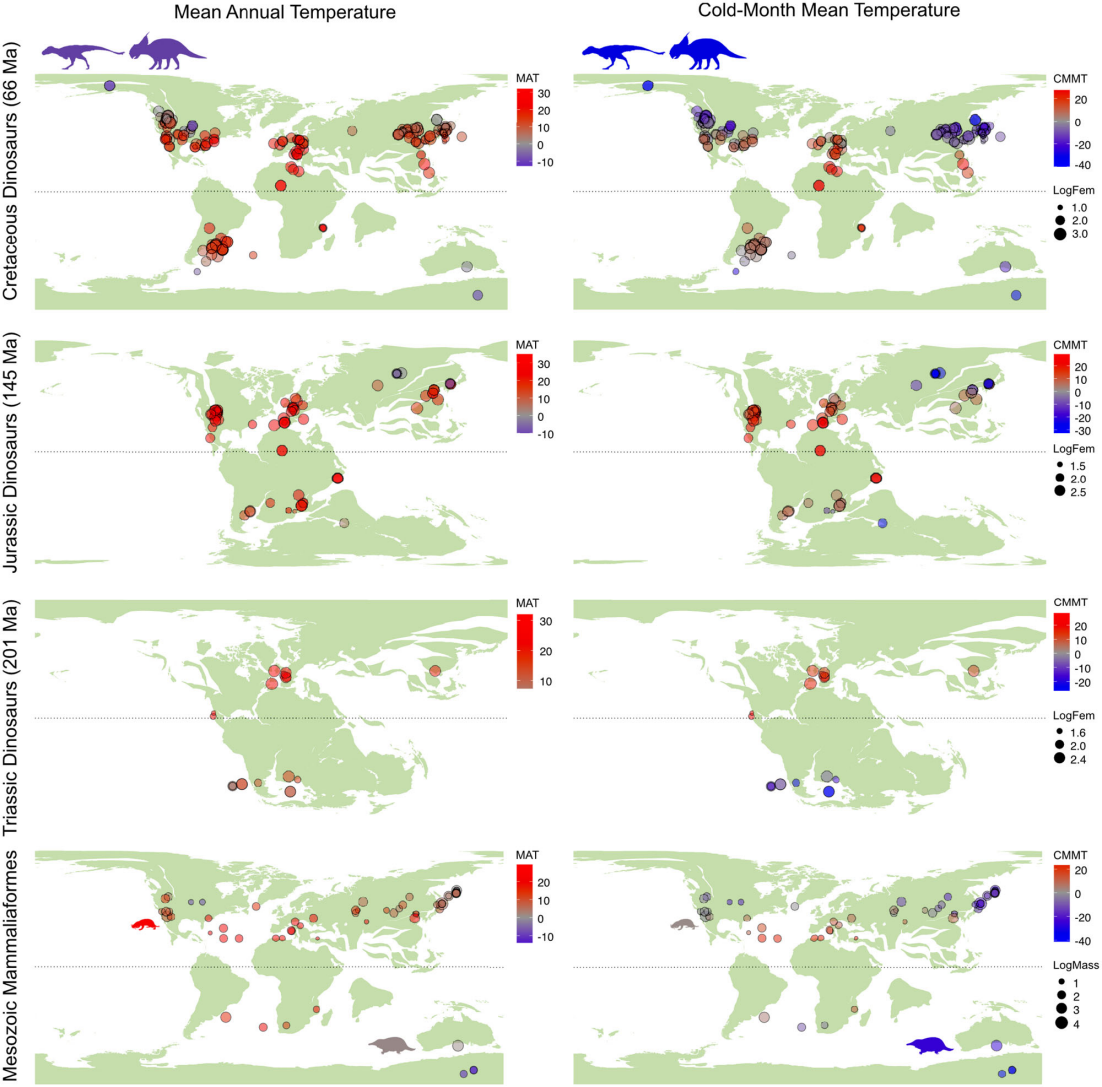
Distribution of body size is not explained by palaeotemperature or palaeolatitude in mammaliaforms or dinosaurs. Top three rows show the geographic distribution of Mesozoic dinosaurs in the Cretaceous, Jurassic, and Triassic. The bottom row shows the geographic distribution of Mesozoic mammaliaforms. Palaeogeographic maps show the locations of fossil taxa, obtained from GPlates using the R package chronosphere^81^, with points scaled by log_10_-transformed body size (millimetres for dinosaurs and grams for mammaliaforms). Colours represent the estimated local mean annual palaeotemperature (left) and cold-month mean palaeotemperature (right) in °C. Silhouettes highlight *Nanuqsaurus* and *Pachyrhinosaurus* of the high-latitude Cretaceous Prince Creek Formation and the Mesozoic mammaliaforms *Morganucodon* and *Steropodon*, coloured by estimated MAT and CMMT (°C). MAT mean annual temperature, CMMT cold-month mean temperature, Ma million years ago, LogFem log_10_-transformed femur circumference, LogMass log_10_-transformed body mass.

We repeated the same approach using estimated local mean annual and cold-month mean temperatures (MAT and CMMT) instead of palaeolatitude. Temperatures were inferred from HadCM3BL-M2.1aD, a general circulation model^31^. No effect of palaeotemperature on dinosaur body size evolution was found (MAT: *p*_MCMC_ = 0.26, median β = −0.0008 (95% CI = −0.003, 0.002), median *R*^2^ = −0.041 (95% CI = −0.061, −0.021); CMMT: p_MCMC_ = 0.32, median β = −0.0004 (95% CI = −0.002, 0.001), median *R*^2^ = −0.043 (95% CI = −0.098, −0.008); Figs. 1–2, Supplementary Table 3, Supplementary Fig. 3). We replicated these results using a smaller dataset of inferred body masses (p_MCMC_ > 0.1; Supplementary Table 3), and a model incorporating an increase in body size through time (Cope’s rule) was not supported over the simplest model (BF = 9.94; Supplementary Table 1).

Next, we analysed estimated body mass data (log_10_ grams) for 62 Mesozoic mammaliaforms using the approach outlined for dinosaurs. Model selection, again, favours a simple model (BF = 9.7–24.57; Supplementary Table 2) that doesn’t support a relationship between body mass and absolute palaeolatitude (*p*_MCMC_ = 0.06, median β = 0.01 (95% CI = −0.002, 0.04), median *R*^2^ = 0.027 (95% CI = −0.051, 0.095); Supplementary Table 3, Supplementary Fig. 2). Estimated local palaeotemperatures were also unassociated with mammaliaform body mass evolution (MAT: *p*_MCMC_ = 0.18, median β = −0.008 (95% CI = −0.02, 0.009), median *R*^2^ = −0.0034 (95% CI = −0.069, 0.058); CMMT: *p*_MCMC_ = 0.07, median β = −0.008 (95% CI = −0.02, 0.003), median *R*^2^ = 0.019 (95% CI = −0.057, 0.09); Supplementary Table 3, Supplementary Fig. 4). As with the dinosaurs, a model incorporating Cope’s rule was not supported (BF = 12.08; Supplementary Table 2). Together, these results are consistent with the expectation that Bergmann’s rule was absent in Mesozoic homeotherms.

Sampling bias is a pervasive challenge for comparative analyses of fossil data^27^. To test whether these biases influenced our modelling results, we developed a geographic- and time-specific sampling metric and included it as a covariate in our regression analyses^32^ (see “Methods” section). Through model selection, we found that the number of tetrapod fossil-bearing formations and occurrences in each geographic region and geologic period did not explain the variation observed in Mesozoic dinosaur or mammaliaform body size (BF = 15.87–22.67; Supplementary Tables 1 and 2). Therefore, sampling biases do not explain body size variation across latitudes or climatic temperatures (Supplementary Fig. 5).

### Assessing Bergmann’s rule in extant birds and mammals

**Fig. 3:**
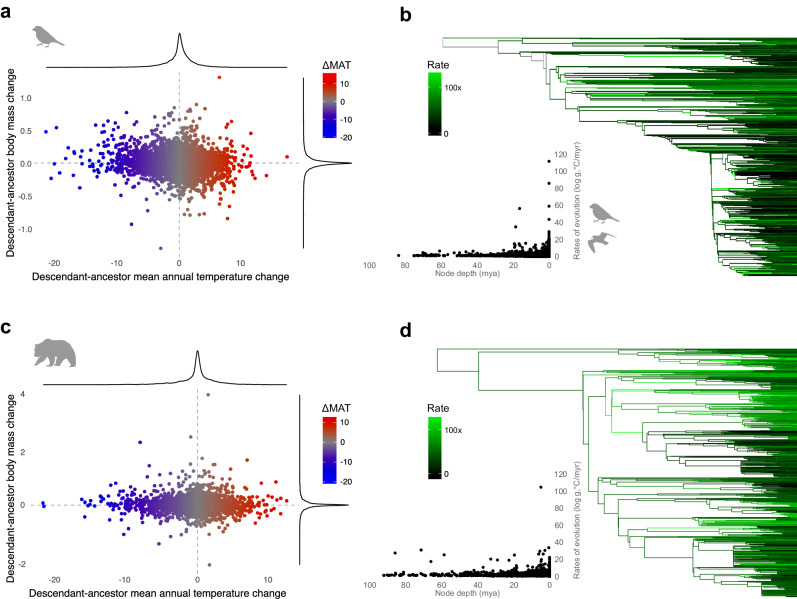
Mean annual temperature and body size evolution among extant birds and mammals. **a** Estimated branch-wise changes in body mass (log_10_ g) as a function of mean annual temperature (°C) along branches of the avian phylogeny. A trend from upper left to lower right would be consistent with Bergmann’s rule. Point colour reflects estimated changes in MAT. Birds are represented by the *Geospiza* silhouette (Ryan Cubo; CC0 1.0 license). **b** Extant bird phylogeny with branches coloured by rates of body mass evolution (log_10_ g/myr), controlling for MAT (°C). Scatter plot shows rates as a function of node height with silhouettes highlighting Darwin’s finches (*Geospiza*) and seabirds (Laridae; Sean McCann; CC0 1.0). **c** Estimated branch-wise changes in body mass (log_10_ g) as a function of mean annual temperature (°C) along branches of the mammalian phylogeny. **d** Extant mammal phylogeny with branches coloured by rates of body mass evolution (log_10_ g/myr), controlling for MAT (°C). Mammals are represented by the *Ursus arctos* silhouette (Tracy Heath; CC0 1.0). g grams, MAT mean annual temperature, mya million years ago, myr million years.

We next analysed 5496 extant bird and 2305 extant terrestrial mammal species (Fig. 3; Supplementary Fig. 6). These two groups are descended from the Mesozoic dinosaurs and mammaliaforms. As with the fossil analyses, our phylogenetic models accounted for geographic range and climatic temperature variation. We find no support for a relationship between body mass (log_10_ grams) and absolute latitude in birds (*p*_MCMC_ = 0.29, median β = ~0 (95% CI = 0, 0.0004), and median *R*^2^ = −0.0002 (95% CI = −0.001, 0.001)) or in mammals (*p*_MCMC_ = 0.11, median β = 0.0008 (95% CI = −0.0004, 0.0018), and median *R*^2^ = 0.001 (95% CI = −0.0021, 0.0064)) (Supplementary Table 3). Applying our approach to temperature instead of latitude, we find a small effect of temperature on body mass in extant birds, as predicted by Bergmann’s rule (p_MCMC_ < 0.001, median β = −0.0036 (95% CI = −0.0041, −0.0032), and median *R*^2^ = 0.13 (95% CI = 0.097, 0.17)). An effect is also found in mammals, though with an *R*^2^ close to zero (p_MCMC_ = 0.004, median β = −0.0025 (95% CI = −0.0043, −0.0007), median *R*^2^ = 0.01 (95% CI = −0.0005, 0.024)). The highest evolutionary rates (>10× the background rate) of temperature-mediated body size are seen in groups that speciated and dispersed widely since the Early Miocene (23 Ma)^33^ (Fig. 3).

## Discussion

The fossil record provides a wealth of unique climatic and biodiversity data unavailable in the modern world, yet it is an underutilised data source for testing macroecological principles^34^. Our palaeotemperature estimates from the HadCM3BL-M2.1aD model show global mean annual temperatures in the Triassic ranged from approximately 30 °C at the equator to 3–10 °C at mid to high latitudes (Fig. 2). Equatorial temperatures were comparable in the Cretaceous; however, temperatures at mid to high latitudes ranged from −11 to 27 °C. Our modelled temperatures are consistent with previously published estimates using the HadCM3BL-M2.1aD model^15,35,36^, though they skew colder than proxy evidence at high latitudes^31,37^.

The Late Cretaceous Prince Creek Formation of Northern Alaska (PCF) is the highest-latitude (80°–85°N palaeolatitude) dinosaur-bearing unit currently known and one of the few exhibiting evidence of freezing temperatures and occasional snowfall^38,39^. The PCF records strong seasonality with an inferred cold-month mean annual temperature of −2.0 ± 3.9 °C from proxy evidence^37,40^, but there is compelling evidence that dinosaurs endured these cold and dark periods and were year-round residents of the Arctic^37,38,41^. However, no evidence exists that dinosaurs found in the PCF were larger compared to related species from lower latitude formations^38^. Dinosaurs from the PCF, representing nine families, are comparable in size to their relatives from more southern Late Cretaceous North American localities^38^ (see Supplementary Discussion). For example, while originally described as a dwarf taxon^42^, our recently collected fossils of *Nanuqsaurus hoglundi*, the only known tyrannosaurid from the PCF, exhibit adult body sizes within the range of lower latitude relatives, such as *Daspletosaurus*^38^ (see Supplementary Discussion). While troodontid teeth from the PCF are larger than those from Montana^43^, body size estimates for the PCF troodontid^42^ are comparable to troodontids from Alberta, such as *Latenivenatrix*^44^ (see Supplementary Discussion).

We find no support for latitude or global climatic temperature shaping body size evolution in dinosaurs and mammaliaforms from the Mesozoic. These findings do not preclude a relationship between climate, physiology, and geographic distribution. For example, no sauropods have been discovered in polar regions, suggesting they may have been poikilothermic^15^. A shift towards warmer climates after the end-Triassic mass extinction may have facilitated their geographic expansion^36^. Although Mesozoic dinosaurs were likely homeothermic ancestrally^45,46^, secondary ectothermy may have evolved in some ornithischians, according to biomolecular evidence^46^, but not others, like *Maiasaura*, according to histological evidence^47^. Further, Jurassic mammaliaforms may have lacked the elevated metabolisms of extant endotherms^48^. Despite this potential variation, we find that latitudinal and temperature effects on body size were absent across Mesozoic dinosaur and mammaliaform groups.

A consensus on Bergmann’s rule has been stymied by variable definitions over time^4,5^. In extant birds and mammals, Bergmann’s rule is speculated to operate at varying taxonomic levels, from intraspecific relationships^49^ to monophyletic groups^4^. Most studies supporting Bergmann’s rule evaluate trends within species^50^ or among assemblages of species^6,51^. Our study tests Bergmann’s rule in a phylogenetic context, where ancestral changes in latitude (or temperature) explain body size evolution. Our approach accounts for evolutionary relatedness by allowing closely related species to be more similar in body size to one another than to distantly related species, as necessitated by the rule^4,5,51^ and in accord with recent research^52–54^. Among extant birds and mammals (the descendants of Mesozoic dinosaurs and mammaliaforms), we do not find an association between body mass and latitude^4^. We find a marginal effect of temperature on the evolution of body mass in mammals – a 1 °C increase in temperature results in a 0.6% decrease in expected body mass (g), but temperature explains essentially no variance in body mass (median *R*^2^ = 0.01, 95% CI = −0.0005, 0.024).

While we find no evidence of latitude influencing the evolution of avian body mass, a small temperature effect is found, concordant with Bergmann’s rule and recent studies^52–54^, and which may be mitigated in part by nest structure and migration^54^. Our models show that a 1 °C decrease in temperature results in a 0.8% increase in expected avian body mass (g). Temperature explains a modest amount of body mass variation (median *R*^2^ = 0.13, 95% CI = 0.097, 0.17), which is consistent with a climatic temperature version of Bergmann’s rule (a moderate effect is expected given the myriad genetic and environmental factors known to influence body mass). The highest rates (>10×) of avian body mass evolution, accounting for temperature, are seen in groups that speciated and dispersed widely since the Early Miocene (23 Ma)^33^, such as the globally distributed seabirds in the Laridae^55^ and the recently radiated *Geospiza*^56^ (Fig. 3). We find no evidence for a temperature effect in Mesozoic birds (Avialae), which suggests that body size evolution and biogeography in modern birds may have been influenced by Bergmann’s rule during Cenozoic climatic change. This is also consistent with a decrease in avian body size associated with anthropogenic global warming^17,18^.

Macroecological rules provide fundamental insights into how ecosystems function, how species coexist and interact, and how biodiversity is maintained. They also strongly influence our strategies for managing biodiversity during an age of climate change because latitudinal gradients of body size have been hypothesised to impact extinction risk^57^. Extending such rules into deep time opens pathways to evaluate their validity and broaden their impact. For example, dinosaurs and mammals had independent origins in the Mesozoic, under a globally warmer climate regime. Our models of Bergmann’s rule harness these deep time data, starting in the Triassic, to calibrate expectations for their descendants in the Present across 251 million years of evolution. We find that body size evolution during the Mesozoic radiations of mammals and dinosaurs were not associated with dispersal to cooler climates. Moreover, homeothermy evolved independently in these two groups and our results suggest that this adaptation, without ancillary changes to body mass, allowed mammals and birds to succeed in habitats spanning global latitudinal gradients. In sum, our results provide a unique perspective on body size evolution in extant homeotherms and highlight the importance of fossil data for evaluating long-held general principles in macroecology.

## Methods

### Mesozoic data

Bergmann’s rule, as originally proposed, operates among closely related taxa^3–5^ and should have a phylogenetic structure. We used phylogenetic regression models to test Bergmann’s rule in Mesozoic dinosaurs and mammaliaforms but focused primarily on the former, given their larger sample size and range in body size. As the foundation for our phylogenetic analyses, we use a comprehensive dinosaur phylogeny from Benson and colleagues^29^, which includes 624 dinosaurian and avemetatarsalian taxa, and a phylogeny of extinct mammaliaforms from Huttenlocker and colleagues^58^. We added *Nanuqsaurus hoglundi* to the phylogeny of Benson et al.^29^ in place of *Teratophoneus* (a close relative in the Benson et al. phylogeny based on results by Brusatte and Carr^59^ and Voris et al.^60^) and time-constrained it to 69.1 Ma based on the average of the most recently reported dates from the Prince Creek Formation.

To test Bergmann’s rule across taxa, we collected femoral circumferences (log_10_ millimetres) and palaeogeographic occurrences for 339 dinosaur species from the datasets of Benson and colleagues^29^ and O’Donovan and colleagues^25^. We added femur circumference estimates for recently collected specimens of *Nanuqsaurus*, housed at the University of Alaska Museum of the North Earth Science Collection (UAMES) (see Supplementary Materials for an extended discussion on new information for *Nanuqsaurus*). Femoral circumference was used as a proxy for body size via the conventions from Benson and colleagues^29^. Our femoral circumference data were supplemented with a smaller dataset of imputed body masses (*n* = 319) from Benson and colleagues^29^. O’Donovan and colleagues^25^ originally obtained the palaeogeographic data from the Paleobiology Database (PBDB), which converts the present-day latitudes and longitudes of fossil sites into palaeolatitude and palaeolongitude values using GPlates software (https://www.gplates.org/). To ensure our palaeogeographic locations were estimated consistently, we re-rotated the modern-day occurrences using a coupled Atmosphere-Ocean General Circulation Model, HadCM3L-M2.1aD^31^ (more information below). We also obtained body mass data (log_10_ grams) for 62 Mesozoic mammaliaforms from Slater and colleagues^61^ and palaeogeographic occurrences for each species from the PBDB and applied the same rotation corrections with HadCM3BL-M2.1aD.

The disproportionate sampling of fossils in different geographic regions has been shown to influence comparative analyses of diversification and geographic dispersal. It is conceivable that the known variation in body size is correlated with the number of fossil-bearing rock formations in a particular region and point in time. To test for such an effect on our regression results, we followed Gardner and colleagues’ approach^35^ and collected the number of unique tetrapod fossil-bearing rock formations across multiple geographic zones. Rather than the broad geographic regions used by Gardner and colleagues, we collected formation counts across nine 20-degree latitudinal zones (Supplementary Fig. 5). We further subdivided these geographic-specific formation counts into the three Mesozoic geologic periods, the Triassic, Jurassic, and Cretaceous. Based on a protocol by Dunne and colleagues^35^, we also calculated the number of terrestrial tetrapod occurrences (removing taxonomically unidentifiable fossils and those not based on body fossils) for the same time- and geographic-specific zones. Using tetrapod-wide occurrences allows us to approximate a given taxon’s ‘true absence’ (e.g., if a dinosaur species was absent in a specific latitudinal zone but other tetrapods were present). Based on their average age and palaeolatitude, we assigned each taxon a geographic- and time-specific formation and occurrence count as additional independent variables in our regression analyses. The full list of occurrences and geologic formations used, along with their ages and palaeocoordinates, are provided in the supplementary materials.

### Palaeotemperature models

Palaeotemperature data were inferred using an updated version of the UKMO HadCM3 family, a coupled Atmosphere-Ocean General Circulation Model (AOGCM), with a lower resolution ocean component (specifically HadCM3BL-M2.1aD, following the nomenclature of Valdes et al.^31^). Both the atmosphere and ocean component models have a resolution of 3.75° longitude × 2.5° latitude, with 19 hybrid levels in the atmosphere, 20 vertical levels in the ocean, and equations solved on the Arakawa B-grid with sub-grid scale processes (e.g., as convection, cloud, orographic variance terms) parameterised. It is essential to run deep time simulations from long integrations to allow full equilibrium simulations so that the climate is fully representative of the time-specific boundary conditions (topography, bathymetry). Because of these long integration periods, we implement a freshwater flux adjustment scheme by adding freshwater to prevent salinity drift and balance the water loss from inland drainage basins over millennia. This is negligible over short periods; however, it is required to equilibrate the model over longer periods to prevent ocean salinity estimates from becoming unrealistically saline. Sea ice is calculated on a zero-layer model with possible partial sea ice coverage and a consistent salinity assumed for ice. The model has a further update that includes modifications to cloud condensation nuclei density and cloud droplet effective radius, following the work of Sagoo et al.^62^ and Kiehl and Shields^63^. This produces warmer higher latitude temperatures where previous models are too cool (Cold-Pole Paradox) compared to proxy data and reproduces a pre-industrial climate without modification.

Because geological data recording land surface vegetation for Triassic – Cretaceous geologic stages are uncertain and globally sparse, we use a version of the model that includes the dynamical vegetation model TRIFFID (Top-Down Representation of Interactive Foliage and Flora Including Dynamics) and land surface scheme MOSES 2.1^64^. TRIFFID predicts the distribution and properties of global vegetation based on plant functional types (PFTs) in the form of fractional coverage (and thus PFT co-existence) within a grid-cell based on competition equations of climate tolerance of five plant functional types. The ocean model is that of Cox^65^, a fully three-dimensional, full primitive equation model. HadCM3BL-M2.1aD can reproduce the modern climate^31^ and has actively contributed to the Coupled Model Intercomparison Projects (CMIP3-5) as well as Palaeomodel Intercomparison Model Projects (PMIP1-4).

Thirty model simulations cover each geologic stage of the Jurassic – Cretaceous, each comprising unique stage-specific palaeogeographic boundary conditions (topography, bathymetry and land ice where relevant; see Farnsworth et al.^66^ for palaeogeographic reconstruction) from Getech Plc. All atmospheric constituents (CH_4_, N_2_O, CFC-11, CFC-12, CFC-113, HCFC-22, HFC-125, HFC-13p4A, SO_4_-aerosol, O_3_) except *p*CO_2_ are modelled at pre-industrial levels. *p*CO_2_ concentrations are set at the stage level for each simulation based on the stage mid-point from the Foster et al.^67^ reconstruction, except for the Maastrichtian, which was from the Rae et al.^68^ compilation (dashed line; Supplementary Fig. 7) due to the over-reliance on stomatal CO_2_ reconstructions in the Foster et al.^67^ dataset for the Maastrichtian.

The solar constant was based on Gough^69^. Each stage-specific DEM is interpolated from a 0.5° × 0.5° grid onto the model 3.75° × 2.5° grid. Surface soil conditions were set at a uniform medium loam everywhere, as stage-specific global soil parameters during the Triassic – Cretaceous are unknown. All other boundary conditions (such as orbital parameters, volcanic aerosol concentrations, etc.) are held constant at pre-industrial values. To ensure all simulations are fully equilibrated, we use (1) the globally and volume-integrated annual mean ocean temperature trend of less than 1 °C per 1000 years, (2) trends in surface air temperature that are less than 0.3 °C per 1000 years, and (3) a net energy balance at the top of the atmosphere, averaged over 100 years at the end of the simulation, that is less than 0.25/W m^2^. In practice, this means each stage-specific simulation has been run for at least 10,000 model years, often longer. Climate means were produced from the last 100 years of each simulation.

### Extant data

We collected body masses (log_10_ grams), and latitudinal and local environmental temperature ranges for 5496 extant birds^53^. The avian phylogeny was obtained from TimeTree 5^70^. One species from every sister-taxon pair with identical mass, latitude, and temperature values was removed. We also collected body masses (log_10_ grams), and latitudinal and local environmental temperature ranges for 2305 extant mammals, after randomly removing one species in a sister-taxon pair with identical values. Body mass data were sourced from the PanTHERIA database^71^, with additional latitudinal data for *Ursus maritimus* (polar bear) from the southern Beaufort Sea (averaged across three decades)^72^. Latitudinal and local temperature data were sourced from Rolland and colleagues^73^, who originally collected the biogeographic data from the Global Biodiversity Information Facility (www.gbif.org) and the International Union for Conservation of Nature’s Red List website (www.iucnredlist.org). Rolland and colleagues^73^ obtained the temperatures of each occurrence using the mean annual temperature climatic layer (BIO1) from WorldClim (www.worldclim.org). The mammal phylogeny we used from Rolland et al. was originally sourced from Bininda-Emonds et al.^74^.

### Interspecific regression analyses

We conducted Bayesian phylogenetic generalised least squares regressions using log_10_-transformed femur circumference or body mass as the dependent variable. We used the absolute value of latitude as our primary independent variable, which combines data from the northern and southern hemispheres. For our Mesozoic dinosaur models, we also ran models of increasing complexity that included dummy-coded indicator variables for hemisphere location (northern or southern hemisphere), geologic period (Triassic, Jurassic, or Cretaceous), and clade (Theropoda, Sauropodomorpha, and Ornithischia), as well as their interactions with absolute palaeolatitude as explanatory variables. These indicator variables let us test for a difference in the effect of palaeolatitude on body size across space, time, and taxonomic groups. For Mesozoic mammals, due to small sample size in the Triassic (*n* = 2), we only tested for a difference in effect between hemispheres. We also tested if absolute palaeolatitude explains body size after accounting for an increase in body size through time (Cope’s rule) by including the tip ages of species as an additional explanatory variable. We compared the fit of each model by calculating Bayes factors (BF) from the estimated log marginal likelihoods, where a BF > 2 is considered good evidence for the model with the higher marginal likelihood. We selected the model with the highest log marginal likelihood and assessed the statistical support for each regression coefficient by calculating the proportion of slope (*β*) parameter estimates that crossed a value of 0 (p_MCMC_). A low p_MCMC_ means that a considerable proportion of the slope estimates deviates from a flat line. After model selection, we assessed the assumptions of equal variance and normality while accounting for phylogenetic non-independence.

We used BayesTraits V4 to conduct our interspecific regression analyses (https://www.evolution.reading.ac.uk/BayesTraitsV4.0.0/BayesTraitsV4.0.0.html). Mesozoic analyses ran for 12,500,000 iterations with a 2,500,000-iteration burn-in and sampling frequency of 1000. All extant analyses ran for 150,000,000 iterations with a 100,000,000-iteration burn-in and sampling frequency of 1000. We estimated log marginal likelihoods using the Stepping Stone algorithm^75^ with 100 stones sampled every 1000 iterations for the Mesozoic analyses and 500 stones sampled every 1000 iterations for the extant analyses. In addition, we used a Bayesian reversible-jump Markov-chain Monte Carlo procedure to sample a distribution of values for taxa with multiple body sizes, geographic occurrences, and local environmental temperatures using the ‘DistData’ command in BayesTraits. We also estimated phylogenetic signal in the data using Pagel’s lambda; a lambda of 1 indicates high phylogenetic signal.

We ensured that our additional independent variables (occurrence count, hemisphere, geological period, and clade) did not carry redundant information (i.e., multicollinearity) with absolute latitude by calculating variance inflation factors (VIFs) using the package car^76^ in R. There is significant multicollinearity if two or more variables share a VIF > 10.0. Using the R package nlme^77^, we ran maximum likelihood phylogenetic generalised least squares regression models with all independent variables studied for Mesozoic dinosaurs and mammaliaforms. Following our multiple regression protocol, we treated hemisphere, geological period, and clade as “dummy-coded” indicator variables. Northern hemisphere occurrences were coded as 0, and southern occurrences were coded as 1. In our Mesozoic dinosaur models, we coded variables with three categories, like geological period and clade, with two indicator variables, treating the Triassic and Ornithischia as our “baseline” groups. We used the full models to assess multicollinearity. We found that no independent variable showed significant multicollinearity with absolute latitude (Mesozoic dinosaur VIFs: absolute latitude = 1.53, occurrence count = 5.29, hemisphere = 2.80, Jurassic = 4.13, Cretaceous = 5.46, Sauropod = 1.92, Theropod = 1.41; Mesozoic mammaliaform VIFs: absolute latitude = 1.03, occurrence count = 1.23, hemisphere = 1.25).

### Rates of evolution and branch-wise changes

The study of ecological rules, like Bergmann’s rule, demands an account of evolutionary rate variation. Studies have demonstrated substantial variation in rates of body size evolution and geographic dispersal across mammals^19,20^ and avialan and non-avialan dinosaurs^25,29^. To test Bergmann’s rule while accounting for varying rates of evolution, we leveraged a variable rates extension to the phylogenetic independent contrast regression model^78^. This model uses a Bayesian reversible-jump MCMC algorithm to propose shifts in the rate of evolution across a phylogeny under a regression model framework. Under the model, traits evolve by Brownian Motion, and shifts in evolutionary rate are inferred based on deviations in residual variance unpredicted by Brownian Motion. The model proposes rate scalars that adjust individual branch lengths and entire clades such that the residuals meet the expectations of Brownian Motion. Rate shifts relative to the background rate can be identified in reference to the original time-calibrated tree without prior specification as to their location or magnitude within the phylogeny. We tested these variable-rate regression models against those that assume a homogenous rate of evolution by comparing the log marginal likelihoods of both models with Bayes factors. We found good evidence for variable rates of evolution across all taxonomic groups (BF > 4; Supplementary Table 3).

To visualise the magnitude and direction of ancestral changes in body size, absolute latitude, and local environmental temperature, we calculated the contrasts between each sister branch across the extant bird, mammal, and Mesozoic dinosaur and mammaliaform trees, while accounting for variable rates of evolution (Supplementary Fig. 1). To calculate the contrasts, we first conducted univariate variable rates analyses separately for body size, absolute latitude, and local temperature, while randomly sampling multiple occurrences, as described in the interspecific regression tests above. These analyses produced a posterior distribution of trees scaled by the rates of evolution along each branch. We then used the rate-scaled maximum clade credibility trees from the variable-rate analysis on each trait and estimated the maximum likelihood ancestral states for body size, absolute latitude, and temperature using the function fastAnc in the R package phytools^79^. We calculated the contrasts for each branch by calculating the difference between the ancestral state and the immediate descendant. These contrasts represent the ancestral changes in body size, absolute latitude, and temperature. The correlation of these contrasts is equivalent to our phylogenetic regression analyses described above. By plotting the relationship between the contrasts for body size and those for latitude and temperature, we can visualise directionally similar shifts in these variables. To verify this approach, we plotted the estimated contrasts from simulated positive and negative controls (Supplementary Fig. 1). For the negative control, we simulated the independent evolution of two traits for 2500 taxa under Brownian Motion using fastBM in phytools^79^. The distribution of the estimated contrasts for the two traits are unassociated, as expected of independent evolution. The positive control was simulated using a multivariate model of correlated evolution. The distribution of estimated contrasts results in a linear relationship, where the change in one trait along a phylogenetic branch explains that of the second trait. We used the rTraitMult function in the R package ape^80^ to simulate continuous correlated evolution.

Ursidae (bears) provides a clear example of what Bergmann’s rule would look like if it were found to operate across Mammalia (Supplementary Fig. 1) using this plotting scheme. We confirmed an interspecific relationship between body mass and absolute mid-range latitude among eight ursids using Bayesian phylogenetic generalised least squares (median β = 0.0097, median *R*^2^ = 0.75, *p*-value = 0.0027). This amounts to a 25% relative increase in expected body mass (g) with a 1° increase in latitude, where absolute latitude explains 75% of body mass variation across the clade. The greatest positive co-directional changes occur in the common ancestor of *Ursus arctos* (brown bear) and *U. maritimus* (polar bear) and along the terminal branch to *U. maritimus*. We also see the greatest negative co-directional change along the branch to the Southeast Asian *Helarctos melayanus* (sun bear).

### Reporting summary

Further information on research design is available in the Nature Portfolio Reporting Summary linked to this article.

### Supplementary information

Supplementary Information

Peer Review File

Reporting Summary

## Data Availability

All data used in this study, including source data for Figs. 1–3 and Supplementary Figs. 1–7, are accessible in the supplementary materials reposited at Zenodo (10.5281/zenodo.10455929).

## Code availability

All code used in this study, including code for making Figs. 1–3 and Supplementary Figs. 1–7, is accessible in the supplementary materials reposited at Zenodo (10.5281/zenodo.10455929).
