# Supplementary material for: Global latitudinal gradients and the evolution of body size in dinosaurs and mammals
Source: Nat Commun. 2024 Apr 5;15:2864. doi: 10.1038/s41467-024-46843-2 (PMC10997647; doi:10.1038/s41467-024-46843-2)
Supplement: Supplementary file 1 — Supplementary Information [file 41467_2024_46843_MOESM1_ESM.pdf]

Supplementary Materials

Global latitudinal gradients and the evolution of body size in dinosaurs and mammals

Table of Contents	Page
**Supplementary Discussion**	
Extended discussion of Prince Creek Formation fauna body sizes	2
New size estimates for *Nanuqsaurus hoglundi*	2
**Supplementary Tables**	
Supplementary Table 1. Model selection results for the Mesozoic dinosaur analyses	3
Supplementary Table 2. Model selection results for the Mesozoic mammal analyses	3
Supplementary Table 3. Results of the final variable-rates regression models for Mesozoic dinosaurs and mammals, and extant birds and mammals	3
**Supplementary Figures**	
Supplementary Figure 1. Expectation of Bergmann's rule on ancestral changes in body size and absolute latitude	5
Supplementary Figure 2. Mesozoic dinosaur and mammaliaform body size does not covary with palaeolatitude	6
Supplementary Figure 3. Mesozoic dinosaur body size does not covary with palaeotemperature	7
Supplementary Figure 4. Mesozoic mammaliaform body size does not covary with palaeotemperature	8
Supplementary Figure 5. Gaps in the fossil record do not influence the latitudinal distribution of body size	9
Supplementary Figure 6. Body size evolution among extant birds and mammals in relation to latitude and climatic temperature	10
Supplementary Figure 7. Carbon dioxide reconstruction for each target Global Climate Model simulation	11
**Supplementary References**	11

Supplementary Discussion

Extended discussion of the Prince Creek Formation fauna body sizes

Teeth of the Prince Creek Formation troodontid are significantly larger than known taxa from more southern formations (maximum tooth length = 14.3 mm)[1], including specimens from the Two Medicine Formation of Montana (maximum tooth length = 9.53 mm, MOR 553 and TM-088; D. Varricchio, personal communication, July 21, 2023). However, body size estimates for the PCF troodontid predict a height of approximately two meters[1,2], comparable to troodontids from Alberta, such as *Latenivenatrix,* with an estimated height of 1.8 m[1]. Further, in the initial description of the large teeth, it was explicitly stated that invoking Bergmann's rule as an explanation would be speculative at best[1]. This agrees with our findings that if Bergmann's rule is to be considered a general ecological principle, it cannot be applied to only specific taxa. In contrast, all other dromaeosaurid teeth are comparable in size to those from more southerly formations. Other dinosaurs from the Prince Creek Formation, representing nine families, including tyrannosaurid and ceratopsid dinosaurs, are comparable in size to their relatives from more southern Late Cretaceous North American localities[3]. Additionally, *Pachyrhinosaurus perotorum* is known from multiple partial adult skulls[4,5], all smaller than adult skulls of *Pachyrhinosaurus canadensis* from southern Alberta. An unnamed leptoceratopsid has teeth comparable in size to *Leptoceratops gracilis* from the Scollard Formation of Alberta and an unnamed thescelosaurid has teeth comparable in size to *Parksosaurus warreni* from the Horseshoe Canyon Formation of Alberta [3]. Together, these fossils show that almost all dinosaurs in high-latitude climates had body sizes like relatives at lower latitudes. There is no evidence of Bergmann's rule operating on these cold-weather enduring taxa.

New size estimates for Nanuqsaurus hoglundi

Perry[6] conducted a quantitative assessment of the adult body size of *N. hoglundi* via direct proportional scaling of recently collected fossils, housed at the University of Alaska Museum of the North Earth Science Collection (UAMES), to several tyrannosaurid taxa (*Tyrannosaurus rex*, *Daspletosaurus torosus*, *Daspletosaurus horneri*, *Thanatotheristes degrootorum*, *Albertosaurus sarcophagus*, *Gorgosaurus libratus*). Given the similar overall morphology among these tyrannosaurids, these direct scaling exercises are considered appropriate estimates of body size based on available evidence. A list of elements selected for scaling exercises and the measurements used can be found in the Supplementary Mesozoic Data Excel file. Perry scaled the material with tyrannosaurid specimens of known skull length, as there is a greater availability of complete skulls compared to complete skeletons. Additionally, skull length is easily regressed to obtain reliable body lengths[7]. Using these scaling exercises, Perry estimated a range of skull lengths dominantly between 860 and 900 mm. These skull lengths were then used in regression equations to obtain an estimated femur length. Using a skull length of 900 mm yields an estimated femur length of 849.147 mm.

Skull length can also be used to predict body mass in theropods. Using a skull length of 900 mm in a regression equation, Perry[6] yielded an estimated body mass of 1903.66 kg. This body mass estimate was then used in regression equations to obtain an estimated femoral circumference. Using 1903.66 kg in these regressions, Perry yielded an estimated femoral circumference of 337.422 mm, which is within the range of tyrannosaurids like *Daspletosaurus* (226–415 mm). We used these estimates in our phylogenetic comparative analyses described in the main text.

Supplementary Table 1. Model selection results for the Mesozoic dinosaur analyses. Each row includes the regression model description (uniform rate), the number of estimated parameters (No. Par.), the estimated log marginal likelihood (mLH), and Bayes factor (BF) compared to Model 1. The number of parameters also includes the y-intercept and Pagel's lambda, the phylogenetic signal parameter.

Model	No. Par.	mLH	BF
1. Femur ~ Absolute Palaeolatitude (AbsLat)	3	-18.13	-
2. Femur ~ Tip age + AbsLat	4	-23.1	9.94
3. Femur ~ AbsLat + Formations	4	-29.36	22.46
4. Femur ~ AbsLat + Occurrences	4	-26.065	15.87
5. Femur ~ AbsLat + Hemisphere	4	-24.83	13.4
6. Femur ~ AbsLat + Hemisphere + AbsLat:Hemisphere	5	-32.79	29.3
7. Femur ~ AbsLat + Jurassic + Cretaceous	5	-33.33	30.4
8. Femur ~ AbsLat + Jurassic + Cretaceous + AbsLat:Jurassic + AbsLat:Cretaceous	7	-36.91	37.56
9. Femur ~ AbsLat + Sauropodomorpha + Theropoda	5	-31.53	26.8
10. Femur ~ AbsLat + Sauropodomorpha + Theropoda + AbsLat:Sauropodomorpha + AbsLat:Theropoda	7	-46.9	57.54

Supplementary Table 2. Model selection results for the Mesozoic mammal analyses. Each row includes the regression model description (uniform rate), the number of estimated parameters (No. Par.), the estimated log marginal likelihood (Lh), and Bayes factor (BF) compared to Model 1. The number of parameters also includes the y-intercept and Pagel's lambda, the phylogenetic signal parameter.

Model	No. Par.	mLH	BF
1. Body Mass ~ Absolute Palaeolatitude (AbsLat)	3	-79.85	-
2. Body Mass ~ Tip age + AbsLat	4	-85.89	12.08
3. Body Mass ~ AbsLat + Formations	4	-91.19	22.67
4. Body Mass ~ AbsLat + Occurrences	4	-90.38	21.06
5. Body Mass ~ AbsLat + Hemisphere	4	-84.70	9.70
6. Body Mass ~ AbsLat + Hemisphere + AbsLat:Hemisphere	5	-92.14	24.57

Supplementary Table 3. Results of the final variable-rates regression models for Mesozoic dinosaurs and mammals, and extant birds and mammals. Each row includes the sample size of each analysis (N), the Bayes factor support for variable rates over a single-rate model (BF), the median estimated slope (β_1) for the effect of absolute latitude (AbsLat), mean annual temperature (MAT), and cold month mean temperature (CMMT) and associated Bayesian credible interval (95% CI), the proportion of slope estimates that crossed a value of 0 (p_{MCMC}), and the median estimated coefficient of determination (R^2) and associated 95% CI.

Group	N	BF	β_1 (95% CI)	p_{MCMC}	R^2 (95% CI)
Dinosaurs (Femur ~ AbsLat)	339	71.41	0.0009 (-0.0007, 0.002)	0.13	-0.039 (-0.059, -0.02)
Dinosaurs (Femur ~ MAT)	339	56.24	-0.0008 (-0.003, 0.002)	0.26	-0.041 (-0.061, -0.021)
Dinosaurs (Femur ~ CMMT)	339	59.23	-0.0004 (-0.002, 0.001)	0.32	-0.043 (-0.098, -0.008)
Dinosaurs (Mass ~ AbsLat)	319	53.14	0.003 (-0.008, 0.01)	0.17	-0.044 (-0.066, -0.024)
Dinosaurs (Mass ~ MAT)	319	55.22	-0.002 (-0.009, 0.005)	0.29	-0.046 (-0.066, -0.026)
Dinosaurs (Mass ~ CMMT)	319	56.66	-0.0008 (-0.006, 0.004)	0.36	-0.047 (-0.067, -0.029)
Mammaliaforms (Mass ~ AbsLat)	62	8.76	0.01 (-0.002, 0.04)	0.06	0.027 (-0.051, 0.095)
Mammaliaforms (Mass ~ MAT)	62	9.34	-0.008 (-0.02, 0.009)	0.18	-0.0034 (-0.069, 0.058)
Mammaliaforms (Mass ~ CMMT)	62	4.41	-0.008 (-0.02, 0.003)	0.07	0.019 (-0.057, 0.09)
Extant birds (Mass ~ AbsLat)	5496	2122.17	0.00 (0.00, 0.0004)	0.29	-0.0002 (-0.001, 0.001)
Extant birds (Mass ~ MAT)	5496	1834.35	-0.0036 (-0.0041, -0.0032)	<0.001	0.13 (0.097, 0.17)
Extant mammals (Mass ~ AbsLat)	2305	466.91	0.0008 (-0.0004, 0.0018)	0.11	0.001 (-0.0021, 0.0064)
Extant mammals (Mass ~ MAT)	2305	427.79	-0.0025 (-0.0043, -0.0007)	0.004	0.01 (-0.0005, 0.024)

Supplementary Figure 1. Expectation of Bergmann's rule on ancestral changes in body size and absolute latitude. a, Changes in two traits along phylogenetic branches under simulated positive and negative controls. Negative control represents two independently evolving traits. Positive control was simulated using a model of directional correlated evolution, as expected from Bergmann's rule. **b**, Example with Ursidae on how branch-specific changes are calculated (left) from species tip values ($T_{t\#}$) and estimated node values ($T_{n\#}$), along with expected correlation under Bergmann's rule (right). **c,** Estimated branch-specific changes in body mass ($\log_{10}$ g) and absolute latitude among eight ursids. *Ursus americanus*, *U. arctos*, *U. malayanus* (*Helarctos*), and *U. maritimus* silhouettes by Tracy Heath (CC0 1.0).

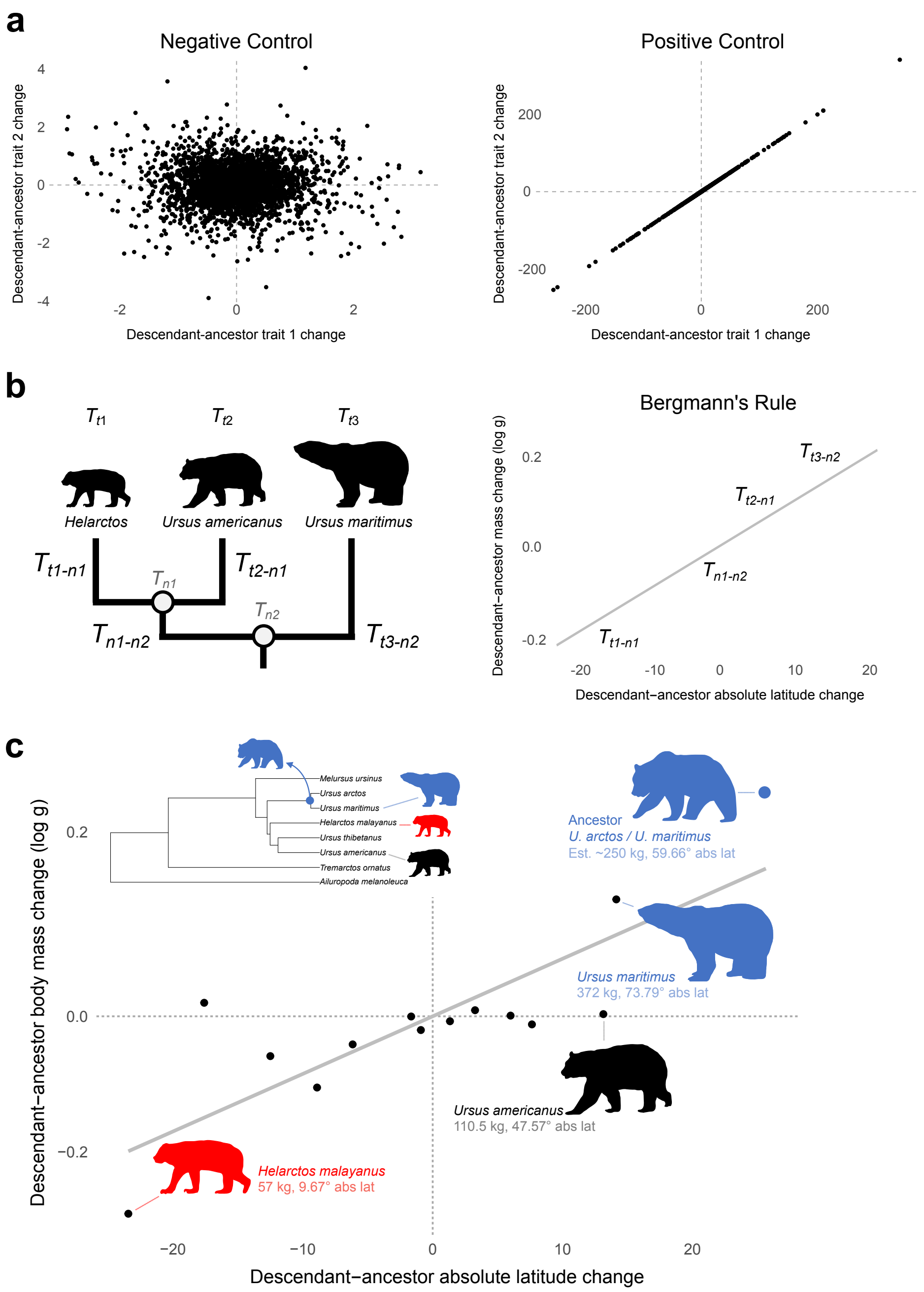

Supplementary Figure 2. Mesozoic dinosaur and mammaliaform body size does not covary with palaeolatitude. a, Femur circumference ($\log_{10}$ mm) as a function of absolute palaeolatitude in 339 Mesozoic dinosaurs. Silhouettes highlight *Spinophorosaurus* (blue; Remes K. et al.; CC BY-SA 3.0) and *Nanuqsaurus* (green; Jaime Headden; CC BY 3.0). **b**, Estimated branch-specific changes in femur circumference ($\log_{10}$ mm) as a function of estimated branch-specific changes in absolute palaeolatitude. Overlapping density plots indicate the distribution of estimated branch-specific changes for each geological period, distinguished by color. **c**, Body mass ($\log_{10}$ g) as a function of absolute palaeolatitude in 62 Mesozoic mammaliaforms. Silhouettes highlight *Morganucodon* (blue; Michael B. H.; CC BY-SA 3.0) and *Steropodon* (green; Nobu Tamura; vectorized by T. Michael Keesey; CC BY 3.0). Posterior (gray) and average (black) regression lines were derived from the variable-rates phylogenetic generalized least squares regression models (p_{MCMC} > 0.05; Supplementary Table 3). Axes labels represent the minimum, 25% quantile, median, 75% quantile, and maximum values.

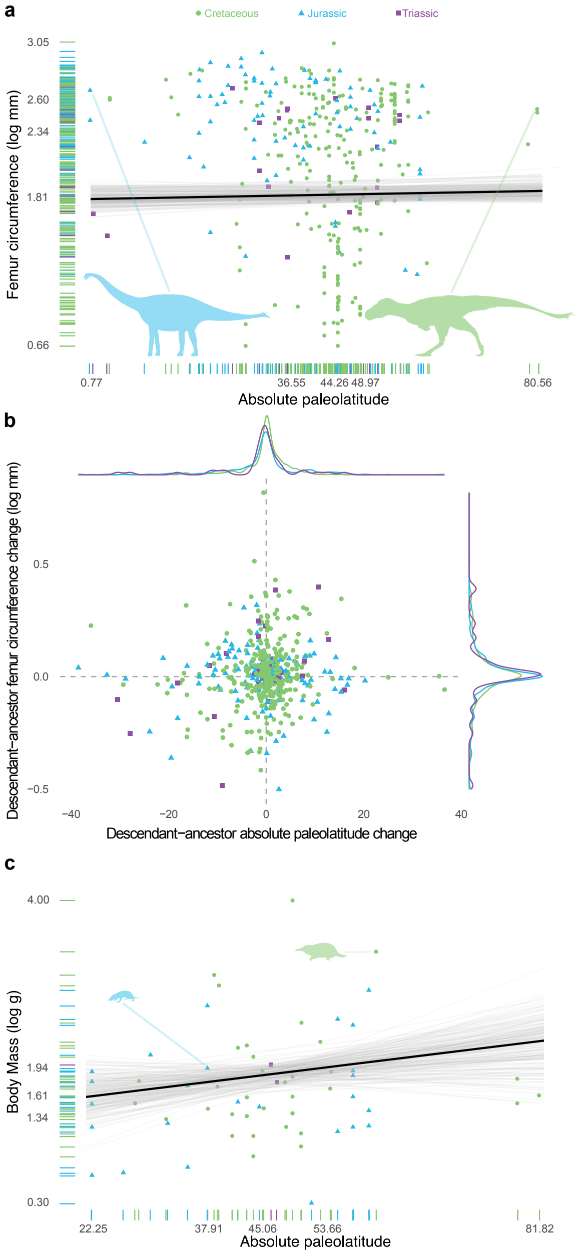

Supplementary Figure 3. Mesozoic dinosaur body size does not covary with palaeotemperature. Femur circumference ($\log_{10}$ mm) as a function of mean annual and cold month mean temperature in 339 Mesozoic dinosaurs, and the estimated branch-specific changes in femur circumference as a function of estimated branch-specific changes in palaeotemperature. Overlapping density plots indicate the distribution of estimated branch-specific changes for each geological period, distinguished by color. Posterior (gray) and average (black) regression lines were derived from the variable-rates phylogenetic generalized least squares regression models ($p_{MCMC} > 0.05$; Supplementary Table 3). Axes labels represent the minimum, 25% quantile, median, 75% quantile, and maximum values.

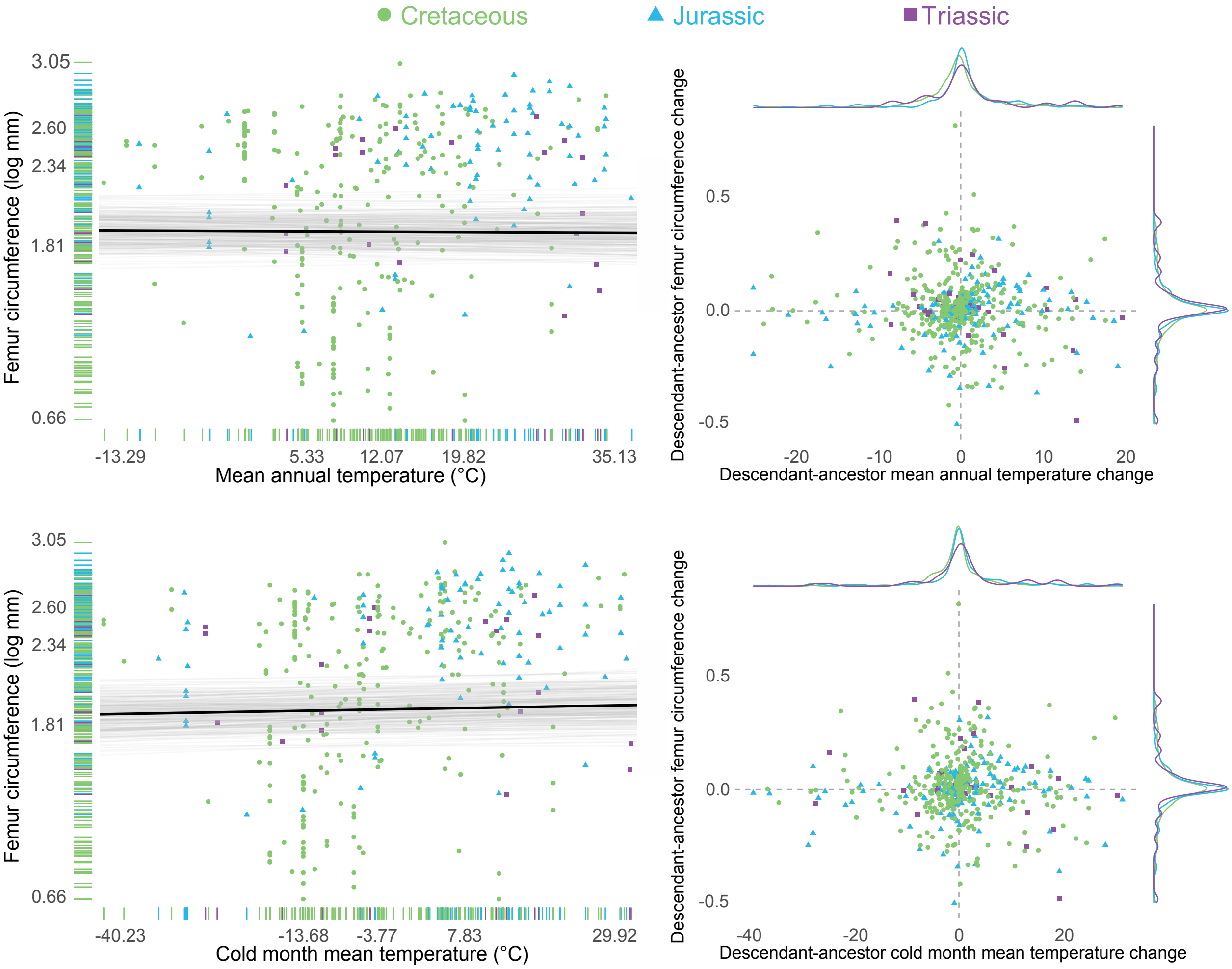

Supplementary Figure 4. Mesozoic mammaliaform body size does not covary with palaeotemperature. Body mass (log_{10} g) as a function of mean annual and cold month mean temperature in 62 Mesozoic mammaliaforms, and the estimated branch-specific changes in mass as a function of estimated branch-specific changes in palaeotemperature. Colors indicate the three Mesozoic geological periods. Posterior (gray) and average (black) regression lines were derived from the variable-rates phylogenetic generalized least squares regression models (p_{MCMC} > 0.05; Supplementary Table 3). Axes labels represent the minimum, 25% quantile, median, 75% quantile, and maximum values.

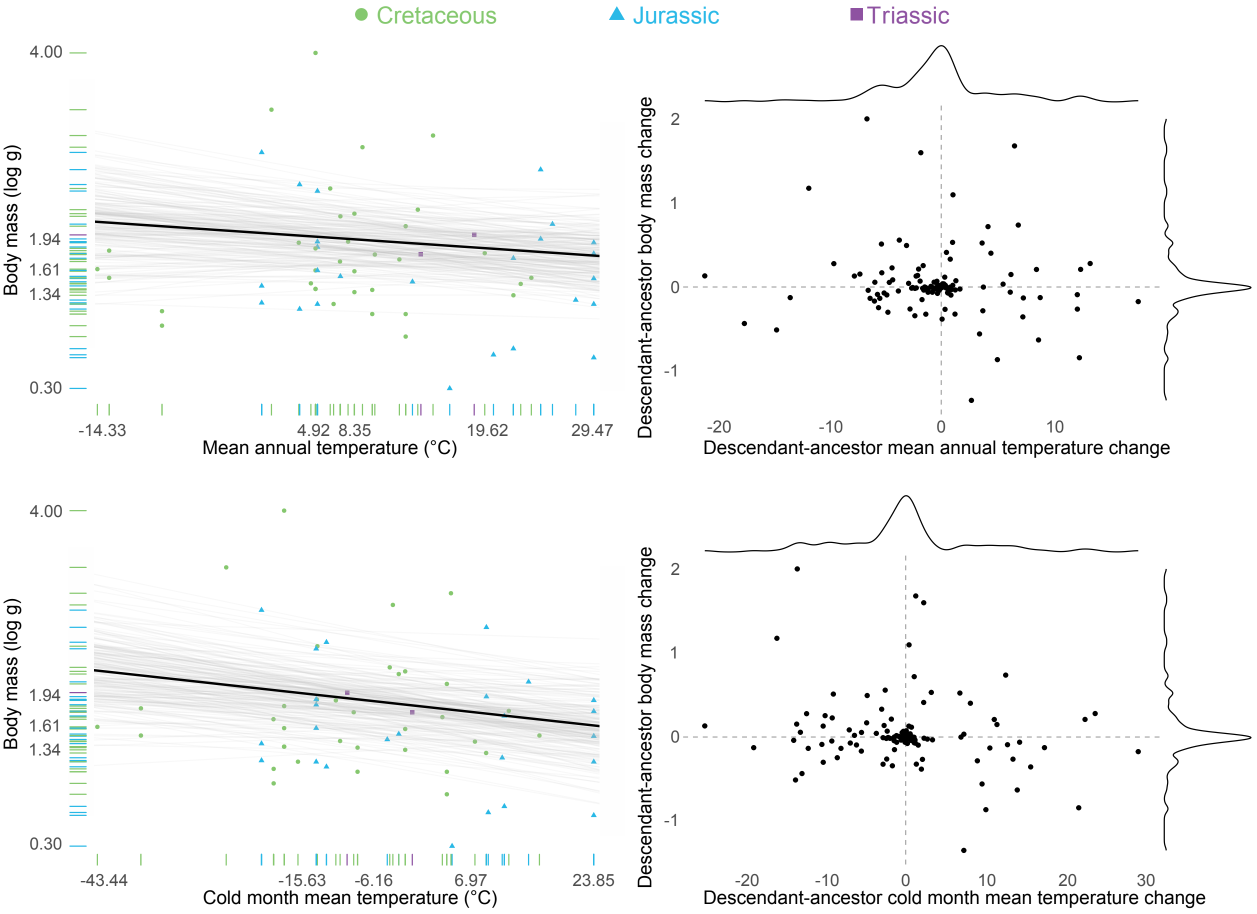

Supplementary Figure 5. Gaps in the fossil record do not influence the latitudinal distribution of body size. Top three rows show the geographic distribution of Mesozoic dinosaurs in the Cretaceous, Jurassic, and Triassic. The bottom row shows the geographic distribution of Mesozoic mammaliaforms. Left two columns show the distribution of body size ranges (maximum - minimum) and occurrence counts for each of the nine latitudinal regions. Colors of occurrence count bars match colors in the map. Palaeogeographic maps show the locations of fossil taxa and were obtained from GPlates using the R package chronosphere[8]. Scatter plots relate logged body sizes of taxa as a function of occurrence count. Posterior (grey) and average (black) regression lines were derived from phylogenetic generalized least squares regressions ($p_{MCMC} > 0.05$). The y-axes represent the minimum, 25% quantile, median, 75% quantile, and maximum values.

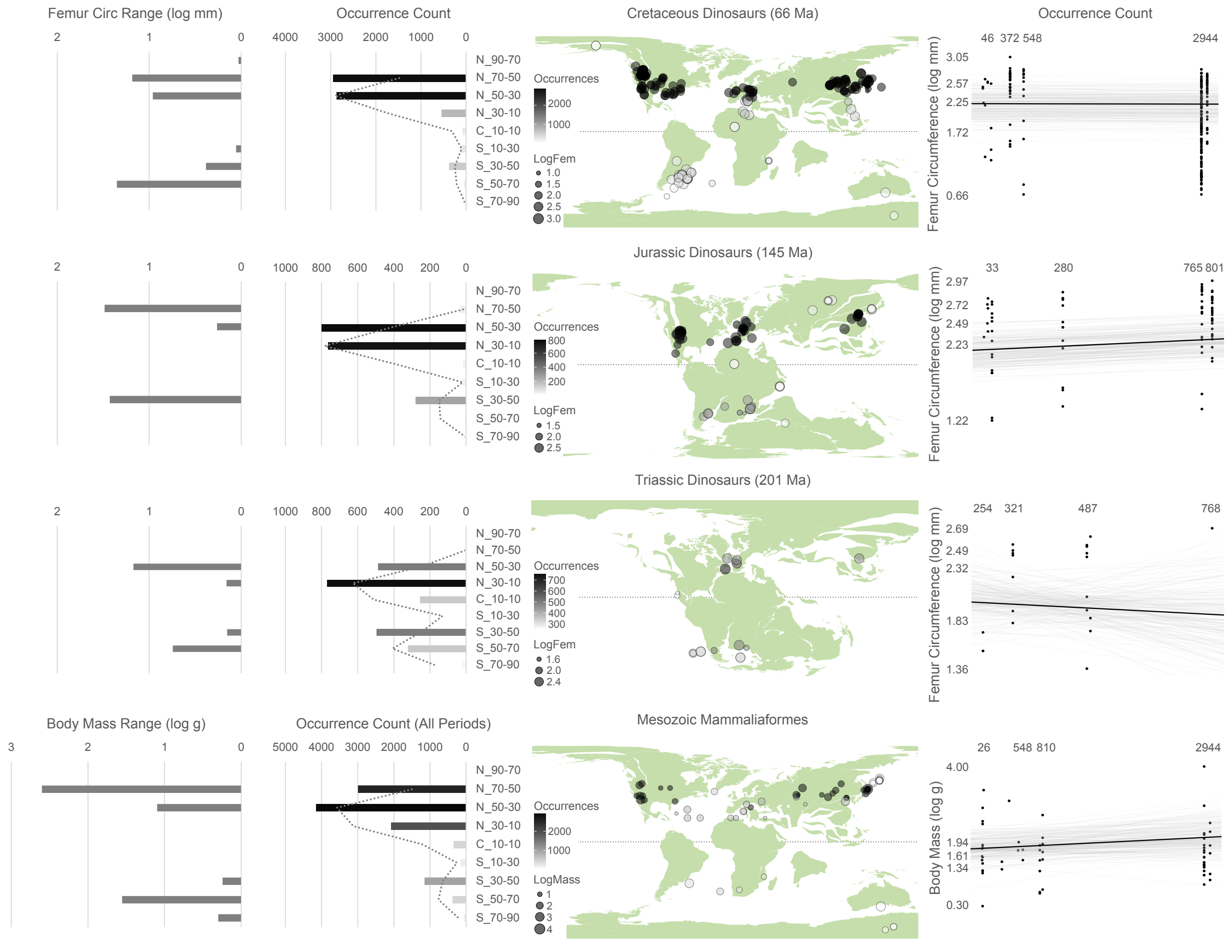

Supplementary Figure 6. Body size evolution among extant birds and mammals in relation to latitude and climatic temperature. a, b, Body mass (log_{10} g) as a function of absolute latitude and mean annual temperature (MAT) among 5,496 extant birds. **c,d,** Body mass (log_{10} g) as a function of absolute latitude and MAT among 2,305 extant mammals. The black best-fit lines are an average of the posterior distribution of regression model estimates (p_{MCMC} > 0.05 for absolute latitude and < 0.05 for mean annual temperature; Supplementary Table 3). Axes labels represent the minimum, 25% quantile, median, 75% quantile, and maximum values.

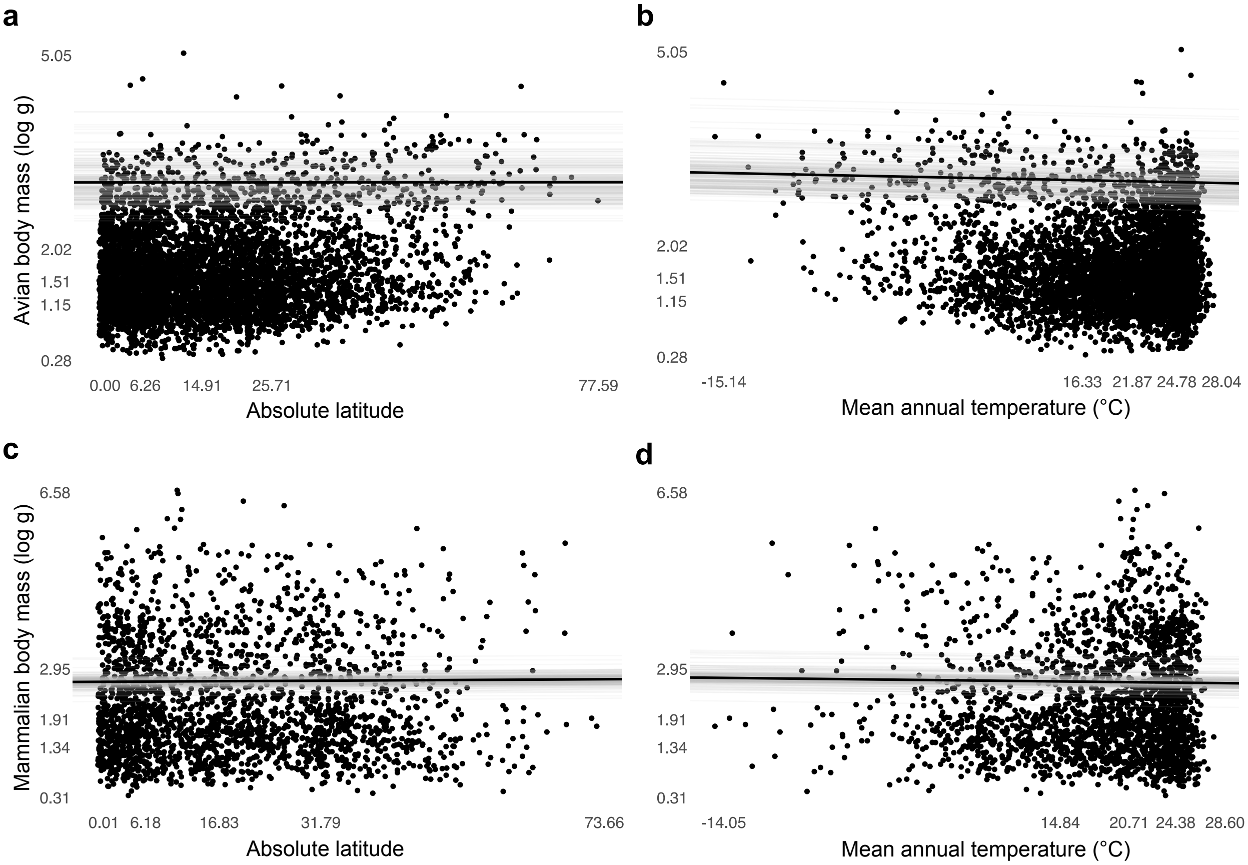

Supplementary Figure 7. Carbon dioxide reconstruction for each target Global Climate Model simulation. Red symbols represent each Mesozoic geologic stage. The blue curve signifies the long-term mean *p*CO_2 reconstructed by Foster et al.[9]. with a variable reconstruction for the Maastrichtian from Rae et al.[10] depicted by the dashed line. Abbreviations: Myrs BP, millions of years before present; ppmv, parts per million volume.

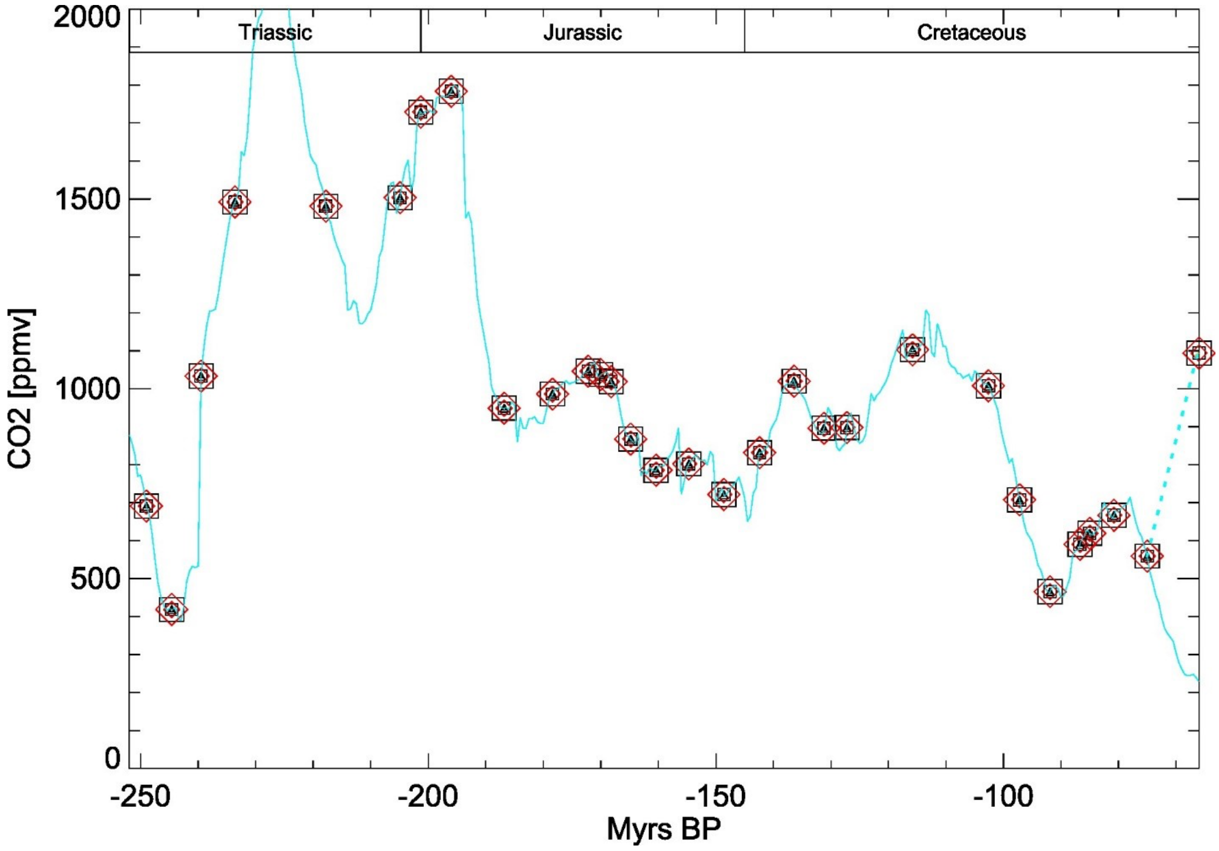

Supplementary References

1. Fiorillo, A. R. On the occurrence of exceptionally large teeth of Troodon (Dinosauria: Saurischia) from the Late Cretaceous of Northern Alaska. *PALAIOS* **23**, 322–328 (2008).
2. Druckenmiller, P. S., Erickson, G. M., Brinkman, D., Brown, C. M. & Eberle, J. J. Nesting at extreme polar latitudes by non-avian dinosaurs. *Curr. Biol.* **31**, 3469-3478.e5 (2021).
3. Tykoski, R. S., Fiorillo, A. R. & Chiba, K. New data and diagnosis for the Arctic ceratopsid dinosaur Pachyrhinosaurus perotorum. *Journal of Systematic Palaeontology* **17**, 1397–1416 (2019).

4. Therrien, F. & Henderson, D. M. My theropod is bigger than yours... or not: estimating body size from skull length in theropods. *Journal of Vertebrate Paleontology* **27**, 108–115 (2007).
5. Currie, P. J. Allometric growth in tyrannosaurids (Dinosauria: Theropoda) from the Upper Cretaceous of North America and Asia. *Canadian Journal of Earth Sciences* **40**, 651–665 (2003).
6. Perry, Z. R. A reinterpretation of Nanuqsaurus hoglundi (Tyrannosauridae) from the late cretaceous Prince Creek formation, northern Alaska. (University of Alaska, Fairbanks, 2023).
7. Benson, R. B. J. *et al.* Rates of Dinosaur Body Mass Evolution Indicate 170 Million Years of Sustained Ecological Innovation on the Avian Stem Lineage. *PLoS Biol* **12**, e1001853 (2014).
8. Kocsis, Á. T. & Raja, N. B. chronosphere: Earth system history variables. https://doi.org/10.5281/zenodo.3530703 (2020).
9. Foster, G. L., Royer, D. L. & Lunt, D. J. Future climate forcing potentially without precedent in the last 420 million years. *Nat. Commun.* **8**, 14845 (2017).
10. Rae, J. W. B. *et al.* Atmospheric CO2 over the past 66 million years from marine archives. *Ann. Rev. Earth Pl. Sc.* **49**, 609–641 (2021).
