# Supplementary material for: Global latitudinal gradients and the evolution of body size in dinosaurs and mammals
Source: Nat Commun. 2024 Apr 5;15:2864. doi: 10.1038/s41467-024-46843-2 (PMC10997647; doi:10.1038/s41467-024-46843-2)
Supplement: Supplementary file 3 — Reporting Summary [file 41467_2024_46843_MOESM3_ESM.pdf]

Corresponding author(s): Lauren Wilson, Jacob Gardner, Chris Organ

Last updated by author(s): 4 March 2024

Reporting Summary

Nature Portfolio wishes to improve the reproducibility of the work that we publish. This form provides structure for consistency and transparency in reporting. For further information on Nature Portfolio policies, see our Editorial Policies and the Editorial Policy Checklist.

Statistics

For all statistical analyses, confirm that the following items are present in the figure legend, table legend, main text, or Methods section.

n/a	Confirmed	
☐	☒	The exact sample size (*n*) for each experimental group/condition, given as a discrete number and unit of measurement
☐	☒	A statement on whether measurements were taken from distinct samples or whether the same sample was measured repeatedly
☐	☒	The statistical test(s) used AND whether they are one- or two-sided<br>*Only common tests should be described solely by name; describe more complex techniques in the Methods section.*
☐	☒	A description of all covariates tested
☐	☒	A description of any assumptions or corrections, such as tests of normality and adjustment for multiple comparisons
☐	☒	A full description of the statistical parameters including central tendency (e.g. means) or other basic estimates (e.g. regression coefficient) AND variation (e.g. standard deviation) or associated estimates of uncertainty (e.g. confidence intervals)
☐	☒	For null hypothesis testing, the test statistic (e.g. *F*, *t*, *r*) with confidence intervals, effect sizes, degrees of freedom and *P* value noted<br>*Give P values as exact values whenever suitable.*
☐	☒	For Bayesian analysis, information on the choice of priors and Markov chain Monte Carlo settings
☒	☐	For hierarchical and complex designs, identification of the appropriate level for tests and full reporting of outcomes
☒	☐	Estimates of effect sizes (e.g. Cohen's *d*, Pearson's *r*), indicating how they were calculated

Our web collection on statistics for biologists contains articles on many of the points above.

Software and code

Policy information about availability of computer code

Data collection	GPlates software through the R package chronosphere was used to collect palaeogeographic maps. The code used to generate these maps can be found in our online supplementary materials reposited at Zenodo (DOI: 10.5281/zenodo.10455929). The coupled Atmospheric Ocean General Circulation Model, HadCM3L-M2.1aD, was used to estimate local palaeotemperatures of fossil occurrence sites.
Data analysis	We used BayesTraits V4 to conduct Bayesian phylogenetic generalised least squares and variable-rate independent contrasts regression analyses (http://www.evolution.reading.ac.uk/BayesTraitsV4.0.0/BayesTraitsV4.0.0.html). The R packages ape, car, nlme, and phytools were used to estimate phylogenetic branch-wise changes in body size, latitude, and temperature and variance inflation factors for our multiple regression analyses. All codes used in this study are accessible in the supplementary materials reposited at Zenodo: DOI: 10.5281/zenodo.10455929.

For manuscripts utilizing custom algorithms or software that are central to the research but not yet described in published literature, software must be made available to editors and reviewers. We strongly encourage code deposition in a community repository (e.g. GitHub). See the Nature Portfolio guidelines for submitting code & software for further information.

Data

Policy information about availability of data

All manuscripts must include a data availability statement. This statement should provide the following information, where applicable:

- Accession codes, unique identifiers, or web links for publicly available datasets
- A description of any restrictions on data availability
- For clinical datasets or third party data, please ensure that the statement adheres to our policy

All data used in this study are accessible in the supplementary materials reposited at Zenodo (DOI: 10.5281/zenodo.10455929). Source data are provided with this paper. Data for Mesozoic mammals and dinosaurs were downloaded from the Palaeobiology Database (PBDB). Data for extant birds were collected from Frohlich et al. 2023. Data for extant mammal analyses were obtained from the PanTHERIA database and Rolland et al. 2018.

Human research participants

Policy information about studies involving human research participants and Sex and Gender in Research.

Reporting on sex and gender	The study did not involve human research participants.
Population characteristics	The study did not involve human research participants.
Recruitment	The study did not involve human research participants.
Ethics oversight	The study did not involve human research participants.

Note that full information on the approval of the study protocol must also be provided in the manuscript.

Field-specific reporting

Please select the one below that is the best fit for your research. If you are not sure, read the appropriate sections before making your selection.

☐ Life sciences ☐ Behavioural & social sciences ☒ Ecological, evolutionary & environmental sciences

For a reference copy of the document with all sections, see nature.com/documents/nr-reporting-summary-flat.pdf

Ecological, evolutionary & environmental sciences study design

All studies must disclose on these points even when the disclosure is negative.

Study description	We developed Bayesian variable-rate phylogenetic regression models to test Bergmann's rule using body size (mass or femur circumference) as the dependent variable and biogeographic and local temperature range data as the independent variables. This was an observational study and, therefore, did not involve treatment factors, an experimental design, or experimental units or replicates.
Research sample	We collected latitudinal occurrences and body sizes for all available extant and extinct dinosaurs and mammals. The sample was aimed to represent all dinosaurs and mammals in the Mesozoic and Present; however, we were limited to sampling taxa that had latitudinal occurrence and body size data.
Sampling strategy	For Mesozoic dinosaurs, a sample size of 339 was determined by taxa in the phylogeny with femur circumference and palaeogeographic data available. For Mesozoic mammals, a sample size of 62 species was determined by taxa present in the phylogeny which also had body mass and palaeogeographic data available. For extant birds, a sample size of 5,496 was determined by taxa present in the bird Time Tree of Life (a topology based on genetic data) which also had body mass, biogeographic, and temperature data available. For extant mammals, a sample size of 2,506 was determined by taxa present in the phylogeny and had body mass, biogeographic, and temperature data available. The extant bird and mammal sample sizes also reflect the random removal of one species from each sister taxon pair with identical trait data.
Data collection	The dinosaur phylogeny, femur circumferences, and palaeogeographic data were collected by Lauren Wilson and Jacob Gardner from Benson et al. (2018), O'Donovan et al. (2018), and the Paleobiology Database (PBDB). Femur circumference data were supplemented with a smaller dataset of imputed body masses from Benson et al. (2018). The Mesozoic mammal phylogeny was obtained from Huttenlocker et al. (2018). Mesozoic mammal body mass and palaeogeographic data were obtained from Slater et al. (2013) and the PBDB. Palaeotemperatures for Mesozoic occurrences were estimated from the palaeogeographic data using HadCM3L, a general circulation climate model. Biogeographic, temperature, and body mass data for extant birds were obtained from Frohlich et al. (2023). The extant avian phylogeny was obtained from TimeTree.org. Extant mammal body masses were obtained from the PanTHERIA database. The extant mammal phylogeny and biogeographic and temperature data were obtained from Rolland et al. 2018.
Timing and spatial scale	NA
Data exclusions	No data were excluded from analyses.

Reproducibility	Phylogenetic comparative analyses were conducted three times to ensure consistent results.
Randomization	NA
Blinding	Blinding is not relevant to our study as it is not experimental in this way.

Did the study involve field work? ☐ Yes ☒ No

Reporting for specific materials, systems and methods

We require information from authors about some types of materials, experimental systems and methods used in many studies. Here, indicate whether each material, system or method listed is relevant to your study. If you are not sure if a list item applies to your research, read the appropriate section before selecting a response.

Materials & experimental systems

n/a	Involved in the study
☒	☐ Antibodies
☒	☐ Eukaryotic cell lines
☒	☐ Palaeontology and archaeology
☒	☐ Animals and other organisms
☒	☐ Clinical data
☒	☐ Dual use research of concern

Methods

n/a	Involved in the study
☒	☐ ChIP-seq
☒	☐ Flow cytometry
☒	☐ MRI-based neuroimaging
